# Where is the patient in the records? Evaluating physiotherapists’ first visit in occupational health primary care pathway for low back pain

**DOI:** 10.1136/bmjoq-2025-003900

**Published:** 2026-02-26

**Authors:** Maija Paukkunen, Birgitta Öberg, Jaro Karppinen, Leena Ala-Mursula, Katja Ryynänen, Riikka Holopainen, Allan Abbott

**Affiliations:** 1 Unit of Physiotherapy, Department of Health, Medicine and Caring Sciences, Linköping University, Linköping, Sweden; 2 Research Unit of Health Sciences and Technology, University of Oulu, Oulu, Finland; 3 Wellbeing Services County of South Karelia, Lappeenranta, Finland; 4 Research Unit of Population Health, University of Oulu, Oulu, Finland; 5 The Wellbeing Services County of North Ostrobothnia Pohde, Oulu, Finland; 6 Wellbeing Services County of South Savo, Mikkeli, Finland; 7 Faculty of Sport and Health Sciences, Jyväskylän Yliopisto, Jyväskylä, Finland; 8 Department of Orthopaedics, Linköping University Hospital, Linköping, Sweden

**Keywords:** Health services research, Patient-centred care, Quality improvement, Implementation science, Occupational Health

## Abstract

**Background:**

Clinical guidelines recommend a biopsychosocial approach to low back pain (LBP) management, with physiotherapists playing a key role in occupational health primary care (OHPC). However, little is known about how their clinical behaviours at the first visit align with guideline-oriented biopsychosocial principles. Therefore, we evaluated LBP management quality in OHPC by applying predefined criteria to physiotherapists’ documentation.

**Methods:**

Based on a cluster-randomised implementation study data (ISRCTN11875357) we analysed 98 electronic patient records (EPRs) documented by 28 physiotherapists across diverse OHPC units. The intervention arm had received 3–7 days of biopsychosocial training. A stratified random sample of EPRs from individuals with LBP was reviewed using a structured researcher’s evaluation tool. Each item was scored dichotomously (yes/no) and evaluated against predefined quality criteria with stepwise thresholds for different work disability risk groups.

**Results:**

Step I, multidimensional biopsychosocial assessment of LBP, was documented in fewer than half of the records (36.5% in the intervention vs 16.7% in the control arm, p=0.081). The biological dimension was well documented in both arms (100% vs 95.8%, p=0.245), while psychological (58.1% vs 25%, p=0.009) and social (54.1% vs 29.2%, p=0.038) dimensions were more frequently documented in the intervention arm.

Step II quality criteria (low-risk patients) were met in 58.1% of intervention versus 4.2% of control records (p<0.001), and step III (medium-risk) in 55.4% versus 4.2% (p<0.001). No EPRs met step IV (high-risk) quality criteria.

The intervention arm more often documented psychosocial assessments, risk stratification, behavioural strategies and advice to stay active. Person-centredness (ie, goals, values, resources, expectations) was rarely documented (36.5% vs 0%, p<0.001).

**Conclusion:**

Training in guideline-oriented biopsychosocial approach was associated with more frequent documentation of behaviours aligned with high-quality LBP management. However, overall quality varied, and person-centred aspects remained underreported. Complementary implementation strategies are required to ensure consistent delivery and documentation of biopsychosocial clinical practice in OHPC.

**WHAT IS ALREADY KNOWN ON THIS TOPIC**

- Low back pain (LBP) is a leading cause of work disability, and occupational health physiotherapists play a key role in its early management. Documentation in electronic patient records (EPRs) reflects delivered care, but no structured evaluation of LBP management quality has been undertaken in this context.

**WHAT THIS STUDY ADDS**

- This study shows that physiotherapists who had received biopsychosocial (BPS) training more consistently assessed psychological and social dimensions of LBP, applied risk stratification and implemented behavioural strategies, while person-centredness and linkage between assessment and treatment planning remained insufficiently documented.

**HOW THIS STUDY MIGHT AFFECT RESEARCH, PRACTICE OR POLICY**

- The study highlights the need for complementary implementation strategies (e.g. structured templates, mentoring and EPR integration to ensure consistent delivery and documentation of BPS dimensions of LBP in occupational health primary care.
- The study provides direction for the development of standardised minimum datasets and adoption of BPS quality indicators as benchmarks for developing national standards in LBP management.

## Introduction

 Low back pain (LBP) is the leading cause of disability worldwide^1^ and a common reason for work absence.^2^ Current understanding recognises LBP as a complex condition influenced by biological, psychological and social factors, all of which influence pain experience and related disability.^1^ Although biopsychosocial (BPS) management is recommended in clinical guidelines, its implementation in occupational health primary care (OHPC) remains limited.^3^

The BPS approach integrates multidimensional assessment and management. The biological dimension targets functional ability (eg, promoting activity, lifestyle changes, short-term pharmacological treatment if necessary). The psychological dimension addresses maladaptive beliefs, reducing fear-avoidance behaviour and psychologically informed intervention, when needed. The social dimension includes, for example, work demands. BPS principles include patient involvement, shared decision-making and interdisciplinary collaboration.^4^,^6^ Risk stratification tools such as the Start Back Tool (SBT)^7 8^ and Örebro Musculoskeletal Pain Screening Questionnaire (ÖMPSQ)^9^ and its short version ÖMPSQ short-form (SF)^10^,^12^ are recommended to identify individuals at higher risk for prolonged LBP-related disability and tailor care accordingly.^7 13^ Also, early dialogue with the workplace is critical to support work participation.^14^ However, there is little consensus on what data should be routinely collected in OHPC.^15^ Currently, electronic patient record (EPR) documentation in work-related musculoskeletal disorders is inconsistent, and no standardised data content requirements exist internationally.^16^

Quality improvement frameworks such as the Plan-Do-Study-Act (PDSA) cycle provide a structured way to implement and evaluate changes in care delivery.^17^ We have previously reported the ‘plan’ and ‘do’ phases of the PDSA cycle, evaluating effectiveness,^18^ costs^19^ and professionals’ experiences^20^ of care after a BPS training programme for the assessment and management of LBP versus usual care. The current ‘study’ phase uses EPR data to evaluate whether physiotherapists’ documentation reflects BPS principles following training. The findings will inform the subsequent ‘act’ phase to further advance quality improvement actions. In this context, it is essential to assess observable clinical behaviours, because changing knowledge or attitudes does not always translate into behaviour change.^21^,^23^ Therefore, it is important to evaluate whether clinical BPS target behaviours, for example, early risk stratification, goal-oriented planning and person-centredness,^20^ are visible in documentation. Routinely collected EPRs may provide a pragmatic source of real-world evidence for evaluating care processes and can be used to measure quality of care.^1624^,^26^ In sum, our aim was to evaluate the quality of LBP management by applying predefined criteria to physiotherapists’ documentation. We hypothesised that the quality of EPRs documented by physiotherapists who had received training in guideline-oriented BPS management would more frequently align with these criteria compared with physiotherapists without such training.

## Methods

### Study design, setting and participants

This study is a secondary analysis of physiotherapists’ EPR data from a two-arm cluster randomised controlled trial (ISRCTN11875357), described in detail elsewhere.^18 27^ Shortly, Finnish occupational health services include statutory preventive activities and, where contracted, OHPC implying primary care level curative care provided by multidisciplinary teams comprising physicians, nurses, physiotherapists and psychologists.^28^

**Figure 1:**
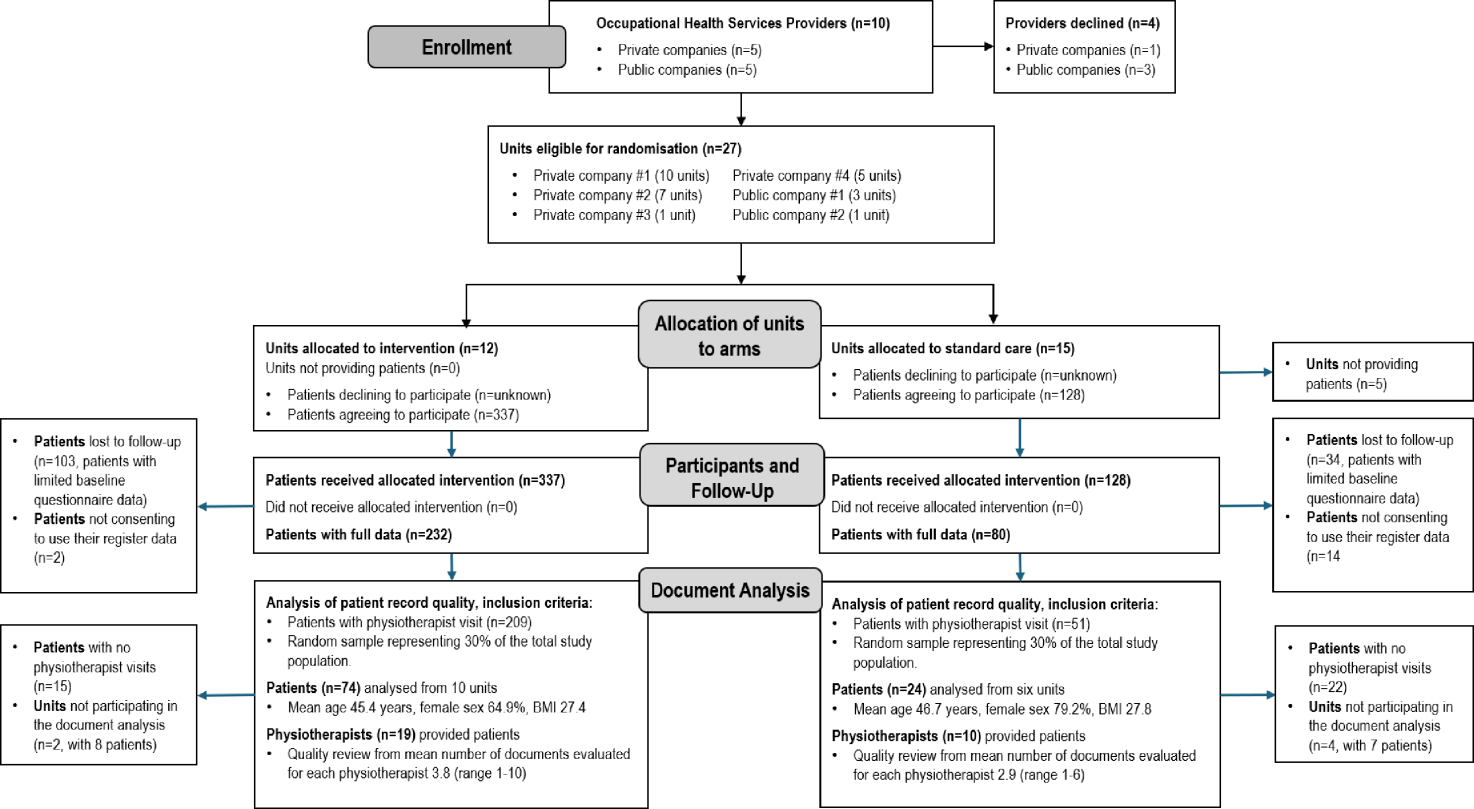
Flow chart of the study. BMI, body mass index.

Six companies providing OHPC (27 units) across Finland participated (figure 1). Units were randomised to intervention or control arms. Between September 2017 and December 2018, physiotherapists and physicians examined eligible patients aged 18–65 years with LBP with or without radicular pain. Exclusion criteria included suspected serious pathology or urgent need for care.^18^ A 30% random sample of EPRs (n=98) was drawn from patients with complete baseline data, consent for register data and at least one physiotherapy visit. Additionally, OHPC providers’ permission for data collection was needed. Random sampling was performed by an independent researcher to ensure proportional representation from the trial arms. Researchers evaluating EPR data, the data abstractors, were blinded to allocation to arms.

### Intervention

The BPS training programme (4-day initial, 3-day booster) included live patient demonstrations, problem solving and role-play.^27 29^ Trainers included experts in pain psychology, rehabilitation medicine and physiotherapy. Participating physiotherapists and physicians disseminated knowledge to their multidisciplinary teams’. Two experienced physiotherapists tutored the study sites.

The participating physiotherapists were trained to apply BPS assessment, use risk stratification tools (SBT, ÖMPSQ-SF) and tailor care to the level of disability risk (low, medium, high). For all patients, the goal was to make the LBP understandable and to focus on modifiable factors according to the individual assessment. Instructions on documentation were not included in the training. Online supplemental figure 1 outlines the BPS guideline used in the training.

The control arm received no such training. The basic education of the use of the BPS approach in assessment and treatment of LBP has been mostly limited.

### Document analysis

An evaluation tool was specifically developed to enable systematic exploratory EPR review to evaluate the quality of physiotherapists’ first visit in OHPC pathway for LBP. Items were derived from the intervention protocol,^27^ clinical BPS target behaviours^20^ (online supplemental figure 1), multidimensional factors associated with disabling LBP,^29^ International Classification of Functioning, Disability and Health model^30^ and quality index study in LBP care.^31^ The three-page tool covered anamnesis (pain characteristics, functional ability, work ability, person-centred aspects), dimensions of assessment (biological, psychological and social) and assessment, whether the documented first-line and second-line treatments aligned with guidelines as well as the low-risk, medium-risk and high-risk profiles. Items were scored using a binary approach (yes=documented, no=not documented) and a single mention in the EPR was sufficient to score an item as documented. The evaluation tool aimed to identify both the aspects physiotherapists documented and those that were omitted, offering insight into the comprehensiveness of LBP management. The detailed description of the evaluation tool and its components is presented in online supplemental table 1.

#### Definition of quality criteria for studying the impact of the intervention

**Figure 2:**
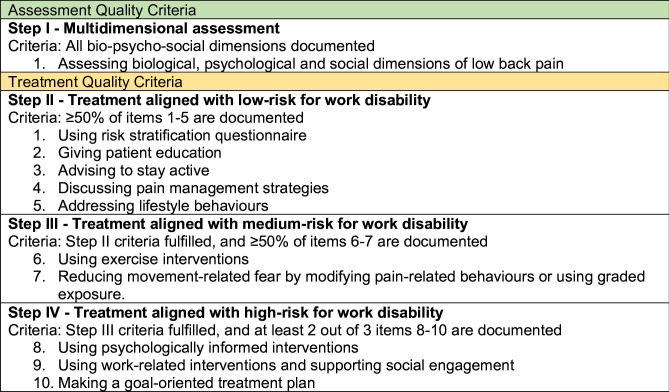
Quality criteria definitions for low back pain management.

The assessment and treatment quality were evaluated using predefined stepwise criteria, starting from reflecting adherence to BPS guideline, *step I* (figure 2). Although each step was considered to represent equally high-quality care, the steps were interpreted in relation to appropriate alignment with the patient’s risk group: *step II* treatment with low risk; *step III* treatment with medium risk and *step IV* treatment with high risk for work disability according to ÖMPSQ-SF.

### Data collection

Data abstraction using the evaluation tool was performed by three researcher pairs (MP and KR, MP and JH, MP and IT) who reviewed the first physiotherapy visit EPR after patient consent. Each EPR was read in full and analysed deductively, using printed evaluation tools. Two researchers jointly reviewed each record and completed the tool by consensus. Discrepancies were resolved through discussion, with a third researcher (JK) consulted when needed. After the first 10 cases, the completed evaluation tools, open questions and uncertainties were reviewed with JK to ensure consistency and clarify interpretations. Altogether, JK was consulted on three occasions for consensus.

Data abstraction was performed by researchers with 10-year to 30-year clinical experience. MP and IT are occupational health physiotherapists. KR and JH are occupational physicians, and KR and JK are specialists in Physical and Rehabilitation Medicine. MP and JK contributed to BPS training but had no affiliation with participating OHPC companies. JH, IT and KR were not involved in training and worked in different companies.

### Baseline characteristics

Characteristics of participating physiotherapists included gender, age, workplace, number of days participating to BPS training and number of EPRs in the sample. Baseline patient data included demographics, general health, LBP characteristics, functional status and work ability as described previously^18^ including age, gender, body mass index (BMI, self-reported weight per squared height kg/m^2^), smoking, self-rated health (EuroQol 5 dimensions (EQ−5D), 0–100 Visual Analogue Scale), health-related quality of life using the EuroQol 5 dimensions 3 levels (EQ-5D-3L), the Depression Scale, pain duration, the Oswestry Disability Index (ODI), work ability (0–10 Numerical Rating Scale), number of sick leave days due to LBP over the preceding 3 months and fear of physical activity or work (Fear-Avoidance Beliefs Questionnaire).

### Statistical analysis

Baseline characteristics are presented as means with SD, medians with IQRs and proportions (%) and counts. The statistical significance of the differences between intervention and control arms was estimated using the independent-samples t-test, χ^2^ or Mann-Whitney U tests, as appropriate.

Evaluation tool items were analysed descriptively using binary scoring (yes/no). Demographic outcomes were summarised as counts and proportions. Differences between arms were tested using Fisher’s exact test. Analyses were conducted using SPSS statistics, V.29 (IBM Corp). A power analysis using Fisher’s exact test (two-tailed, α=0.05, power=0.80, allocation ratio 0.25) indicated that 99 participants (79 in intervention arm, 20 in control arm) were needed to detect a difference in proportions (expected 0.42 vs 0.10).

## Results

EPR data from 98 clinical encounters documented by 28 physiotherapists across 16 OHPC units in five companies were included in the analysis. The majority of physiotherapists were female (78.6%, n=23) with a mean age of 48.1 years (SD 8.3). Of the 19 physiotherapists in the intervention arm, 79% (n=15) had received the BPS training. Four physiotherapists were introduced to the BPS approach through peer dissemination only and had documented five of the EPRs. In the control arm, EPR analysis involved 10 physiotherapists contributed, of whom one had received BPS training and had documented one EPR. The mean number of EPRs evaluated per physiotherapist was 3.8 (range 1–10) in the intervention and 2.9 (range 1–6) in the control arm.

The patient baseline characteristics (n=98) are presented in table 1. The mean age was 45.6 years (SD 9.7), and 68.4% (n=67) were female. Functional limitation was mild 43.9% (n=43) or moderate 45.9% (n=45) according to the ODI. The median EQ-5D index was 0.79 (0.75–0.86). Based on the ÖMPSQ-SF, 41.8% (n=41) of patients were classified to low-risk, 25.5% (n=25) to medium-risk and 32.7% (n=32) to high-risk group. There were no statistically significant differences in baseline characteristics between the intervention (n=74) and control (n=24) arms.

**Table 1:** Sample baseline characteristics

	Total n=98		P value
	Intervention n=74	Control n=24	
General health-related			
Age* (years)	45.4 (9.8)	46.7 (9.7)	0.571
Female sex†	64.9 (48)	79.2 (19)	0.190
Body mass index* (kg/m^2^)	27.4 (4.6)	27.8 (4.3)	0.672
Smoking†	18.9 (14)	16.7 (4)	0.804
Self-rated health status‡ (1–100)	70 (59–85)	73 (65–85)	0.238
EQ5D (0–1)‡	0.79 (0.74–0.86)	0.79 (0.75–0.86)	0.912
DEPS score ‡	7 (2–11)	6.2 (1–8.75)	0.360
LBP-related			
Duration of pain			
<2 weeks†	6.8 (5)	16.7 (4)	0.349
2–11 weeks †	36.5 (27)	29.2 (7)	
>3–12 months †	16.2 (12)	25.0 (6)	
>12 months†	40.5 (30)	29.2 (7)	
Work ability-related			
Work ability ‡ (0–10)	7 (6—8)	6.8 (6–8.75)	0.721
Number of sick leave days due to LBP during preceding 3 months †	48.6 (36)	45.8 (11)	0.221
Number of sick leave days due to LBP during preceding 3 months ‡	7 (3–32.5)	20 (10.5–26)	0.267
Pain-related fear (FABQ)-Work ‡	14.4 (5–22)	13.3 (1.5–22.25)	0.602
Pain-related fear (FABQ)-Physical activity ‡	12 (9–16)	11 (7–16)	0.557
Drug prescriptions †	74.3 (55)	83.3 (20)	0.421
Non-psychotropic drug prescriptions†	67.6 (50)	83.3 (20)	0.195
Psychotropic drug prescriptions†	43.8 (32)	47.8 (11)	0.812
Comorbidities			
Number of comorbidities*	0.81 (1.04)	0.58 (1.1)	0.469
Hypertension†	16.2 (12)	12.5 (3)	0.756
Depression†	16.2 (12)	8.3 (2)	0.507
Migraine†	10.8 (8)	4.2 (1)	0.446
Hypothyroidism†	9.5 (7)	4.2 (1)	0.675
Asthma†	6.8 (5)	8.3 (2)	1
Diabetes†	5.4 (4)	8.3 (2)	0.633
Sleep apnoea†	6.8 (5)	4.2 (1)	1
Obesity†	4.1 (3)	12.5 (3)	0.155

* Mean (SD), p value for between-group difference from independent-samples t-test.

† Percentage (frequency), p value for between-group difference from χ2 test.

‡ Median (IQR), p value for between-group difference from Mann-Whitney U test.

DEPS, Depression Scale; EQ-5D, EuroQol 5 Dimensions 3 Levels score; FABQ, Fear Avoidance Beliefs Questionnaire; LBP, low back pain.

### Quality of anamnesis

*Pain characteristics,* that is, LBP history taking, functional capacity and *work ability* were well documented across both arms. *Pain-related functional behaviours* (ie, communicative or overprotective behaviours such as avoidance of activities) were more frequently documented in the intervention arm (71.6% vs 37.5%, p=0.004). The *triage* process (ie, the screening for red flags) was not systematically documented. *Person-centred aspects,* such as patient’s goals, values, resources and treatment expectations, were documented rarely overall (table 2a).

**Table 2:** Quality of anamnesis and assessment

	Total n=98				P value
	Intervention n=74		Control n=24		
	%	N	%	n	
**(a) Quality of anamnesis**					
Baseline status (documented)					
Pain characteristics					
LBP history taken	86.5	64	79.2	19	0.514
Time course	83.8	62	70.8	17	0.233
Acute	10.8	8	8.3	2	1
Subacute	5.4	4	4.2	1	1
Recurrent	29.7	22	25	6	0.797
Persistent	37.8	28	33.3	8	0.809
Pain area	81.1	60	79.2	19	1
Localised	33.8	25	26	6	0.462
Widespread	47.3	35	54.2	13	0.641
Pain progression	74.3	55	79.2	19	0.787
Improving	18.9	14	50	12	0.006
Stable	14.9	11	8.3	2	0.511
Deteriorating	40.5	30	20.8	5	0.092
Red flags	14.9	11	8.3	2	0.511
Functional capacity					
Activity limitation	83.8	62	79.2	19	0.757
Pain-related functional behaviours	**71.6**	**53**	**37.5**	**9**	**0.004**
Workability	71.6	53	62.5	15	0.449
Person-centred aspects	36.5	27	20.8	5	0.212
Patient’s goals	25.7	19	8.3	2	0.09
Patient’s values	13.5	10	0	0	0.113
Patient’s resources	16.2	12	12.5	3	1
Patient’s treatment expectations	6.8	5	0	0	0.33
**(b) Quality of assessment**					
*Step I*: multidimensional assessment					
Biopsychosocial assessment (all dimensions documented)	36.5	27	16.7	4	0.081
*Biological dimension* (physical and health-related aspects)	100	74	95.8	23	0.245
Physical aspects	98.6	73	95.8	23	0.432
Physical examination	68.9	51	79.2	19	0.439
Range of motions	81.1	60	91.7	22	0.343
Provocative spinal movement directions	64.9	48	70.8	17	0.63
Gait	13.5	10	16.7	4	0.741
Posture	39.2	29	45.8	11	0.636
Motor control	10.8	8	8.3	2	1
Patient specific functions	**37.8**	**28**	**12.5**	**3**	**0.023**
Pain-related physical behaviours	**37.8**	**28**	**12.5**	**3**	**0.023**
Autonomic arousal	6.8	5	4.2	1	1
Muscle strength	17.6	13	16.7	4	1
Muscle activation	5.4	4	4.2	1	1
Physical loading demands	47.3	35	62.5	15	0.243
Work	27	20	45.8	11	0.128
Free time	1.4	1	0	0	1
Tissue hypersensitivity	44.6	33	41.7	10	1
Other physical tests	27	20	41.7	10	0.207
Health-related aspects	86.5	64	83.3	20	0.741
Activity	70.3	52	66.7	16	0.801
Weight/obesity	8.1	6	16.7	4	0.254
Smoking	0	0	4.2	1	0.245
Alcohol abuse	0	0	0	0	
Nutrition	2.7	2	0	0	1
Sleep deficit	41.9	31	33.3	8	0.484
General condition	40.5	30	37.5	9	1
Pain medication	47.3	35	45.8	11	1
*Psychological dimension* (cognitive and emotional aspects)	**58.1**	**43**	**25**	**6**	**0.009**
Cognitive aspects	**45.9**	**34**	**4.2**	**1**	**<0.001**
Pain-related worries	**21.6**	**16**	**0**	**0**	**0.01**
Coping	**29.7**	**22**	**4.2**	**1**	**0.011**
Pain catastrophising	5.4	4	0	0	0.569
Hypervigilance	9.5	7	0	0	0.189
Emotional aspects	43.2	32	25	6	0.149
Distress	12.2	9	12.5	3	1
Low mood	9.5	7	0	0	0.189
Fear of physical activity	**31.1**	**23**	**8.3**	**2**	**0.031**
Anxiety	6.8	5	8.3	2	1
*Social dimension* (social and work-related aspects)	**54.1**	**40**	**29.2**	**7**	**0.038**
Social aspects	25.7	19	12.5	3	0.262
Life-stress events	10.8	8	8.3	2	1
Social support at home	4.1	3	4.2	1	1
Socioeconomic challenges	1.4	1	4.2	1	0.432
Social isolation	4.1	3	4.2	1	1
Cultural background	0	0	0	0	
Visits to several practitioners	9.5	7	0	0	0.189
Complaints seeking	1.4	1	0	0	1
Work-related aspects	47.3	35	25	6	0.061
Workplace issues	10.8	8	12.5	3	1
Work modifications	14.9	11	12.5	3	1
Self-perceived ability to work	**25.7**	**19**	**4.2**	**1**	**0.022**
Fear of not coping with work demands	5.4	4	0	0	0.569
Work community support	6.8	5	0	0	0.33

Bolded values indicate statistically significant difference (p < 0.05)

LBP, low back pain.

### Quality of assessment

*Step I*, documentation addressing all BPS dimensions was found in fewer than half of the EPRs overall (36.5% vs 16.7%, p=0.081, online supplemental figure 2). Biological dimensions were well documented in both arms. Psychological (58.1% vs 25%, p=0.009) and social (54.1% vs 29.2%, p=0.038) dimensions were recorded more frequently in the intervention arm.

#### Biological dimensions

*Physical aspects* were documented for nearly all EPRs in the intervention and control arms (95–98%). These typically included performing physical examination (eg, neurological examination, sit-to-stand, bending, squatting), evaluation of range of motions and identification of provocative spinal movements (ie, directional pain responses). Documentation of physical loading demands, such as occupational, ergonomic, sports or leisure-related activities, was observed in 47–62% of EPRs, with no difference between arms. Assessment of patient specific functions (ie, activities the person was unable to perform or found difficult) was more frequently documented in the intervention arm (37.8% vs 12.5%, p=0.023). Likewise, observations of pain-related physical behaviours (ie, slow guarded movement, avoidance, breath holding, propping with hands, limping, compulsive stretching or use of braces) were documented more often in the intervention arm (37.8% vs 12.5%, p=0.023). Other physical aspects, such as gait, motor control, muscle strength and observation of autonomic arousal (eg, rapid superficial breathing, sweating, agitation), were documented rarely.

*Health-related aspects* were most recorded as general activity levels. In contrast, documentation of smoking, nutrition or substance use was rare. Sleep disturbances, general condition and pain medication use were noted in under half of EPRs.

#### Psychological dimensions

*Cognitive aspects* (eg, worry, coping, catastrophising, hypervigilance) were documented more often in the intervention arm (45.9% vs 4.2% (p=0.001). *Emotional aspects* were also more frequently documented, including fear of physical activity (31.1% vs 8.3%, p=0.031).

#### Social dimensions

*Social aspects* (eg, life stressors, social support, socioeconomic challenges, social isolation, cultural background, complaints-seeking behaviours, multiple consultations) were infrequently documented (25.7% vs 12.5%, p=0.262). *Work-related aspects* (eg, workplace issues, work modifications, perceived ability to work, fear of not coping with work demands, support from work community) were more often addressed in the intervention arm, including perceived work ability (25.7% vs 4.2%, p=0.022). Table 2b details the assessment quality.

### Quality of treatment plans

#### First-line treatments

##### Step II: treatment aligned with low risk for work disability

*Risk stratification* was documented only in the intervention arm (73% vs 0%, p<0.001). *Pain education* and *advice to stay active* were documented more often (32.2% vs 16.7%, p=0.027 and 62.2% vs 12.5%, p<0.001, respectively). Conversely, advice to avoid activity, such as warnings against bending the back, was documented more often in the control arm (4.1% vs 33.3%, p<0.001). *Addressing healthy lifestyle behaviours,* mainly guiding regular physical activities, was more often documented in the intervention arm (41.9% vs 16.7%, p=0.029) while stress reduction, sleep hygiene, substance abandonment and dietary advice were rarely documented. *Pain management strategies* were documented in about half of EPRs.

##### Step III: treatment aligned with medium risk for work disability

Although *therapeutic exercise* was well documented in both arms, the content differed notably. Relaxation and breathing exercises were documented five times more often in the intervention arm (79.7% vs 29.2%, p<0.001). In contrast, postural training (4.1 vs 20.8, p=0.02), postural control training (10.8% vs 33.3%, p=0.022), core exercise (14.9% vs 54.2%, p<0.001) and McKenzie were more frequently documented in the control arm (5.4% vs 20.8%, p=0.037). Muscle strengthening and mobility exercises were documented in 25–42% of cases. Exercise modalities, such as functional restoration, Pilates, muscle endurance, cardiovascular, pelvic floor and balance training, were rarely documented overall.

*Interventions targeting pain-related fear*, such as modifying pain-related behaviour (identifying and practising alternative ways to perform activities and letting go of unhelpful protective habits, eg, guarded movement, avoidance, breath holding, offloading with upper limb support, limping, repetitive stretching and unnecessarily using braces or external supports) and graded exposure to perceived difficult or threatening activities (ie, guiding pain and movement control strategies to build confidence for people to re-engage in valued activities^32^), were more frequently documented in the intervention arm. They were recorded in over half of EPRs (58.1% vs 4.2%, p<0.001), a 14-fold difference.

##### Step IV: treatment aligned with high risk for work disability

Planned *work-related activities* were documented at similar rates at the first visit (33.8% vs 37.5%, p=0.807). These included ergonomics education, work modifications, assistive devices, lifting techniques, activity pacing and promoting physical activity at work. No documentation was found on direct supervisor contact during the visit, or advice to maintain workplace contact during sick leave. *Behavioural interventions,* for example, psychologically informed physiotherapy, were more common in the intervention arm (71.6% vs 4.2%, p<0.001). Relapse planning was rare in the physiotherapist’s first-visit documentation. *Treatment goals* were documented in the majority of EPRs (89.2% overall).

*Link between the anamnesis, assessment and treatment planning*: the EPRs were also evaluated for the presence of patient-identified individual aspects and whether the treatment plan reflected these. Specifically, it was evaluated whether issues identified during the patient interview, such as sleep difficulties, depressive symptoms or pain exacerbation by specific postures or movements, were addressed in the goal setting and treatment plan. A clear trackable, documented alignment between recognising the issue (anamnesis), assessing patient-specific problem and translating into personalised treatment planning was found in only 36.5% of the intervention arm cases and in none of the control arm (p<0.001).

*Second-line treatments*: passive treatment modalities were documented for 45% of EPRs in the intervention arm, and 67% in the control arm, although this difference was not statistically significant. Reported modalities included stretching, nerve mobilisation and massage. However, the use of traction (4.1% vs 20.8%, p=0.02) and passive orthoses (0% vs 12.5%, p=0.013) was more frequently documented in the control arm. Taping, fascia mobilisation and referrals to manual therapies were reported only for a small proportion. Table 3 summarises the treatment quality.

**Table 3:** Quality of treatment plans

	Total n=98				P value
	Intervention n=74		Control n=24		
	%	N	%	N	
First-line treatments					
Step II: treatment aligned with low-risk					
Use of risk stratification tool	**73**	**54**	**0**	**0**	**<0.001**
ÖMPSQ-SF scores	**40.5**	**30**	**0**	**0**	**<0.001**
SBT scores	**48.6**	**36**	**0**	**0**	**<0.001**
Both ÖMPSQ-SF and SBT scores	**24.3**	**18**	**0**	**0**	**<0.001**
Information or education about pain	**32.2**	**32**	**16.7**	**4**	**0.027**
Discussion on imaging	14.9	11	4.2	1	0.283
Advice on return-to-activities	64.9	48	41.7	10	0.057
Advice to stay active	**62.2**	**46**	**12.5**	**3**	**<0.001**
Advice to avoid physical activities	**4.1**	**3**	**33.3**	**8**	**<0.001**
Pain management strategies	58.1	43	45.8	11	0.349
Addressing healthy lifestyle behaviours	**51.4**	**38**	**16.7**	**4**	**0.004**
Sleep hygiene	4.1	3	8.3	2	0.593
Dietary advice	2.7	2	0	0	1
Regular physical activity	**41.9**	**31**	**16.7**	**4**	**0.029**
Substance abandonment	0	0	0	0	
Relaxation or stress reduction	21.6	16	8.3	2	0.225
Step III: treatment aligned with medium-risk					
Use of exercise (therapeutic or patient preference)	93.2	69	100	24	0.33
Physical activity based on patient preference	40.5	30	25	6	0.225
Therapeutic exercise	93.2	69	100	24	0.33
Postural training	**4.1**	**3**	**20.8**	**5**	**0.02**
Postural control training	**10.8**	**8**	**33.3**	**8**	**0.022**
Core exercise or spiral stabilisation	**14.9**	**11**	**54.2**	**13**	**<0.001**
Relaxation or breathing	**79.7**	**59**	**29.2**	**7**	**<0.001**
Muscle strengthening	41.9	31	29.2	7	0.338
Muscle endurance training	2.7	2	0	0	1
Cardiovascular training	2.7	2	0	0	1
Range of movement training	32.4	24	25	6	0.614
Pelvic floor	2.7	2	0	0	1
Balance training	1.4	1	0	0	1
Other	27	20	45.8	11	0.128
Functional restoration	4.1	3	16.7	4	0.059
McKenzie	**5.4**	**4**	**20.8**	**5**	**0.037**
Pilates	6.8	5	8.3	2	1
Reducing pain-related fear	**58.1**	**43**	**4.2**	**1**	**<0.001**
Graded exposure	**47.3**	**35**	**4.2**	**1**	**<0.001**
Modifying pain-related behaviours	**52.7**	**39**	**4.2**	**1**	**<0.001**
Step IV: treatment aligned with high-risk					
Behavioural intervention	**71.6**	**53**	**4.2**	**1**	**<0.001**
Psychologically informed physiotherapy	**73**	**54**	**4.2**	**1**	**<0.001**
Relapse plan	6.8	5	4.2	1	1
Work-related activities	33.8	25	37.5	9	0.807
Information or education on ergonomics	16.2	12	19.2	7	0.233
Work modifications	6.8	5	8.3	2	1
Contact with supervisor during visit	0	0	0	0	
Keep contact with work during sick leave	0	0	0	0	
Recommendation for assistive devices	6.8	5	16.7	4	0.216
Lifting instructions	5.4	4	12.5	3	0.357
Activity pacing	9.5	7	20.8	5	0.16
Physical activities at work	8.1	6	4.2	1	1
Goal-oriented treatment plan					
Treatment plan	93.2	69	91.7	22	1
Clear link between anamnesis, assessment and treatment plan	**36.5**	**27**	**0**	**0**	**<0.001**
Treatment goal	89.2	66	87.5	21	1
Short term	70.3	52	83.3	20	0.289
Long term	27	20	25	6	1
Second-line treatments					
Passive treatment modalities used or advised to	44.6	33	66.7	16	0.099
Joint mobilisation or foam roller	1.4	1	4.2	1	0.432
Joint manipulation	1.4	1	0	0	1
Massage	5.4	4	12.5	3	0.357
Stretching	33.8	25	41.7	10	0.625
Nerve mobilisation	9.5	7	8.3	2	1
Trigger point pressure	0	0	0	0	
Traction	**4.1**	**3**	**20.8**	**5**	**0.02**
Fascia mobilisation	4.1	3	12.5	3	0.155
Heat	0	0	0	0	
Acupuncture	0	0	0	0	
Orthosis	**0**	**0**	**12.5**	**3**	**0.013**
TENS	0	0	0	0	
Taping	5.4	4	4.2	1	1

Bolded values indicate statistically significant difference (p < 0.05)

ÖMPSQ-SF, Örebro Musculoskeletal Pain Screening Questionnaire, short form; SBT, Start Back Tool; TENS, transcutaneous electronic nerve stimulation.

### Quality of stepwise treatment in relation to disability risk

*Step II* (treatment aligned with low risk for work disability) was met in 58.1% (n=43) of EPRs in the intervention arm, compared with 4.2% (n=1) in the control arm (p<0.001). *Step III* (medium-risk) was met in 55.4% (n=41) of EPRs in the intervention arm, compared with 4.2% (n=1) in the control arm (p<0.001). No EPRs met *step IV* (high-risk) criteria.

When analysed by risk groups, 53.3% (n=16) of low-risk patients in the intervention arm met *step II* (low-risk) criteria, and 50.0% (n=15) *step III* (medium-risk) criteria. In the control arm, no EPRs met *step II* (low-risk) criteria (p<0.001).

Of medium-risk patients, 55.6% (n=10) in the intervention arm met *step II* (low-risk) criteria and 50.0% (n=9) *step III* (medium-risk) criteria. In the control arm, 14.3% (n=1) met *step II* (low-risk) criteria and *step III* (medium-risk) criteria (p=0.006).

Of high-risk patients, 65.4% (n=17) in the intervention arm met *step II* (low-risk) criteria *and step III* (medium-risk) criteria. In contrast, 7.7% (n=1) of EPRs in the control arm met these criteria (p=0.001).

### Sensitivity analyses

A sensitivity analysis was performed, excluding patients (n=5) in the intervention group whose physiotherapists received education via peer dissemination; this did not change findings. Similarly, results remained consistent when one patient from the control arm whose physiotherapist had participated in the BPS training was excluded.

## Discussion

The aim of this study was to evaluate the quality of physiotherapists’ LBP management by applying predefined criteria to their EPR documentation and to examine whether their documented clinical behaviours aligned with a guideline-oriented BPS approach. Physiotherapists in the intervention arm more consistently assessed psychological and social dimensions, applied risk stratification and implemented behavioural strategies. However, person-centred aspects were infrequently documented. A clear link between anamnesis, assessment and treatment planning was documented in only one-third of the intervention arm and was absent in the control arm, indicating that treatment planning did not always reflect patient-identified goals and expectations. These results show that although documentation in the intervention arm more often aligned with BPS target behaviours, implementation was partial, underscoring the challenges of translating professional training into consistent clinical behaviours and documentation.

EPRs support informed decision-making and continuity of care.^33 34^ They also safeguard patients’ rights, protect healthcare professionals legally and support quality, safety and continuous learning.^34^,^36^ Although red flag assessment is critical at the first visit, it was rarely documented, possibly reflecting physiotherapy referral pathways and access to national Patient Data Repository. Reassuringly, however, biological dimensions were well covered in the first visits examined in this study. Since 2022, physiotherapists in Finland have served as potential first-contact professionals, underscoring the need to further strengthen first-visit documentation.

OHPC physiotherapists have the potential to positively influence work disability rates, given their access to information on workplace culture, modifications and job demands.^16 37^ While a comprehensive multidimensional assessment and treatment (steps I–III) require considerable time already at the first visit, in OHPC it remains essential to assess the relation between symptoms, work demands and the need for work-related interventions.

Previous research suggests that self-reported clinical behaviours may be overestimated compared with observed behaviours and that physiotherapists should increase their focus on psychosocial factors in practice.^22^ In OHPC, a study on nurses found gaps in the systematic documentation and follow-up of psychosocial stressors, and responses to detected risks were often inadequate.^38^ We observed similar shortcomings: even when psychosocial issues (such as low mood, fear of activity or workplace stressors) were documented, follow-up plans rarely aligned with the patient’s stated concerns and needs.

EPR templates vary across companies.^34^ Most templates in the study included standard sections: consultation, chief complaint (reason for arrival), anamnesis, status and plan. Previous work suggests that loose EPR structures are not well suited for documenting work ability in OHPC.^34^ Consistent with this, we identified gaps in documentation of planned work-related activities. However, our analysis was restricted only to first-visit records. Previous research has also identified poor quality of physiotherapy outcome registration as a barrier to reusing routinely collected data for research purposes.^24 39 40^ A core set of data elements has been proposed for musculoskeletal therapists in OHPC, including health status, pain characteristics, psychosocial and work-related aspects and treatment interventions. As these are potentially predictive of work-related musculoskeletal disorders, there is a need to develop standardised minimum datasets tailored to physiotherapists and physicians in OHPC to support value-based musculoskeletal care.^16^ This aligns directly with the rationale of our study. The structured evaluation tool we applied covered the proposed core content, adding clinical reasoning, functional capacity, work ability and person-centred elements to support informed clinical decision-making and continuity of care. The tool was developed by the research team for retrospective analysis; it was not available for the participating physiotherapists nor included in the BPS training. We found differences between the arms. Our findings suggest an association between having received specific BPS training and more multidimensional structured documentation, as without such training essential BPS dimensions remained undocumented in the control arm.

This study has several strengths. The design allowed for evaluating care in routine OHPC settings without disrupting workflows. Retrospective use of EPRs provided insights into real-world practice, enhancing external validity. Using existing documentation imposed no additional burden on patients or professionals and reduced biases associated with direct observation or interviews. Documentation is legally required in Finland to be completed immediately after the clinical encounter, which enhances accuracy and reliability. The retrospective design enabled timely access to mandated documentation without interfering with patient care. Because registry data are generated independently of research activities, they provide an authentic reflection of clinical behaviour. The cluster-randomised sampling approach ensured representation of the broader Finnish working population and inclusion of individuals across all risk groups supports generalisability. The study complied with ethical and legal requirements, including the General Data Protection Regulation, national data protection legislation^41^,^45^ and ethical approvals. The EPRs contained rich clinical detail, including assessments, treatment decisions, follow-up plans and referrals to multidisciplinary services, providing a comprehensive view of physiotherapy. Two researchers at each time extracted and evaluated the data using a structured evaluation tool, with discrepancies resolved through seeking consensus with a third researcher, strengthening inter-rater reliability and credibility. Predefined quality criteria ensured standardised evaluation, enhancing transparency and reproducibility.

An inherent limitation is that not all aspects of care may have been recorded, while documentation was used as a proxy for physiotherapists’ behaviour. Thus, findings reflect documented rather than all delivered care.^22 46^ Mapping narrative EPRs to predefined criteria required interpretation, introducing the possibility of misjudgement, although a structured evaluation tool was used. Moreover, our scoring approach captured only the presence, not the depth nor adequacy of the documentation. Some EPRs were inconsistently detailed or incomplete, which may have influenced accuracy.^1625 47^,^50^ Contextual factors influencing treatment decisions and continuity of care, such as patient preferences, time constraints or availability of multidisciplinary staff, were generally absent. Important aspects of treatment quality, such as shared decision-making, were seldom recorded in detail.^51^ Variation in EPR systems across providers may have introduced heterogeneity unrelated to clinical practice.^34 52^ No pilot study was undertaken in this secondary analysis of trial data. Some dual roles of researchers may have exposed them to observer bias in EPR abstraction and interpretation, despite efforts to standardise the extraction procedure and apply predefined criteria. To minimise this risk, those involved in the BPS training were not affiliated with OHPC providers, and those employed by participating companies were not involved in the training. Finally, the number of EPR cases (98) was slightly below the calculated size (99 participants required, 79 from intervention, 20 from control arm to detect statistically significant between-group differences), which may have affected statistical power. Transferability to other countries and healthcare systems may be limited by differences in financing, professional scope of practice and documentation infrastructure.

The scope of Finnish employer-funded OHPC differs from general primary care by its close connections to preventive occupational health services including professional consultations, health check-ups and workplace collaboration.^28 53^ Even in OHPC, physiotherapists assess musculoskeletal complaints through clinical examination and functional capacity assessments, evaluate work-relatedness and work ability, provide self-management advice, including exercise guidance and plan follow-up and workplace actions. These role expectations may support more consistent documentation in domains aligned with OHPC processes, whereas other guideline-recommended elements may be documented less systematically if they are not embedded in local workflows, reflected in EPR templates or emphasised during training.

### Implications for healthcare professionals, managers, policy makers and information technologists

Our study provides concrete direction for the ‘act’ phase of the PDSA cycle. First, physiotherapists seem to need additional support to translate BPS principles into clinical behaviours and individualised treatment plans, and including specific training on documentation practices and competency assessment would add to the BPS education.^54^ Second, follow-up mentoring and case-based learning might reinforce the integration of psychological and social dimensions in care, particularly for individuals with higher risk scores.^55^ Third, structured EPRs with BPS target behaviours based on competency checklist as quality indicators could serve as benchmarks for developing national standards in LBP assessment and management. Fourth, integrating patient-reported outcomes and feedback mechanisms would help align care with patient priorities. Structured templates, checklists and EPR prompts could support consistent and systematic documentation of BPS dimensions.

### Implications for researchers

Future research should examine how structured documentation tools, decision-support systems or dialogue-support templates facilitate more consistent and comprehensive implementation of BPS approach. The impact of integrating documentation practices into training, alongside different forms of follow-up support, should be further explored. It would also be valuable to examine whether, and how, adoption of a BPS approach differs in new vs continuous patient-physiotherapist relationships. Furthermore, competency assessment and fidelity testing based on real-life observations should be performed to assess delivery quality in addition to proxy measures. Longitudinal studies are needed to establish whether improved documentation quality translates into better care processes and patient outcomes. Finally, as artificial intelligence (AI) is increasingly used for quality evaluation, documentation must be structured to ensure reliable evaluation. AI scribe applications are in the early adoption phase and may improve documentation efficiency, but also introduce errors of omission, fabrication or substitution. Risks related to privacy and data security must be acknowledged.^56^

## Conclusions

Training in guideline-oriented BPS approach was associated with more frequent documentation of target behaviours consistent with high-quality LBP management. Physiotherapists in the intervention arm more consistently assessed psychological and social dimensions, applied risk stratification and implemented behavioural strategies. However, systematic documentation of psychological and social dimensions, person-centredness and linkage between assessment and treatment planning remained insufficient. These findings underscore the need for complementary implementation strategies to strengthen the consistent delivery and documentation of BPS assessment and management in routine OHPC practice.

## Supplementary material

10.1136/bmjoq-2025-003900 online supplemental file 1

## Data Availability

Data are available upon reasonable request.

## References

[1] Hartvigsen J, Hancock MJ, Kongsted A (2018). What low back pain is and why we need to pay attention. Lancet.

[2] Brus I, Speklé E, Kuijer PP (2022). Occupational recovery of Dutch workers with low back pain. Occup Med (Lond).

[3] Ng W, Slater H, Starcevich C (2021). Barriers and enablers influencing healthcare professionals’ adoption of a biopsychosocial approach to musculoskeletal pain: a systematic review and qualitative evidence synthesis. Pain.

[4] Tagliaferri SD, Miller CT, Owen PJ (2020). Domains of Chronic Low Back Pain and Assessing Treatment Effectiveness: A Clinical Perspective. Pain Pract.

[5] Lin I, Wiles L, Waller R (2020). What does best practice care for musculoskeletal pain look like? Eleven consistent recommendations from high-quality clinical practice guidelines: systematic review. Br J Sports Med.

[6] Gatchel RJ, McGeary DD, McGeary CA (2014). Interdisciplinary chronic pain management: past, present, and future. Am Psychol.

[7] Hill JC, Dunn KM, Lewis M (2008). A primary care back pain screening tool: identifying patient subgroups for initial treatment. Arthritis Rheum.

[8] Hill JC, Whitehurst DGT, Lewis M (2011). Comparison of stratified primary care management for low back pain with current best practice (STarT Back): a randomised controlled trial. Lancet.

[9] Linton SJ, Boersma K (2003). Early identification of patients at risk of developing a persistent back problem: the predictive validity of the Orebro Musculoskeletal Pain Questionnaire. Clin J Pain.

[10] Hill JC, Dunn KM, Main CJ (2010). Subgrouping low back pain: A comparison of the STarT Back Tool with the Örebro Musculoskeletal Pain Screening Questionnaire. Eur J Pain.

[11] Karran EL, McAuley JH, Traeger AC (2017). Can screening instruments accurately determine poor outcome risk in adults with recent onset low back pain? A systematic review and meta-analysis. BMC Med.

[12] Heikkala E, Oura P, Ruokolainen O (2023). The Örebro Musculoskeletal Pain Screening Questionnaire-Short Form and 2-year follow-up of registered work disability. Eur J Public Health.

[13] Linton SJ, Nicholas M, MacDonald S (2011). Development of a Short Form of the Örebro Musculoskeletal Pain Screening Questionnaire. Spine (Phila Pa 1986).

[14] Sennehed CP, Holmberg S, Axén I (2018). Early workplace dialogue in physiotherapy practice improved work ability at 1-year follow-up-WorkUp, a randomised controlled trial in primary care. Pain.

[15] Sullivan V, Wilson MN, Gross DP (2022). Expectations for Return to Work Predict Return to Work in Workers with Low Back Pain: An Individual Participant Data (IPD) Meta-Analysis. J Occup Rehabil.

[16] Wassell M, Vitiello A, Butler-Henderson K (2024). Electronic Health Records for Predicting Outcomes to Work-Related Musculoskeletal Disorders: A Scoping Review. J Occup Rehabil.

[17] Gillam S, Siriwardena AN (2013). Frameworks for improvement: clinical audit, the plan-do-study-act cycle and significant event audit. Qual Prim Care.

[18] Ryynänen K, Oura P, Simula A-S (2021). Effectiveness of training in guideline-oriented biopsychosocial management of low-back pain in occupational health services – a cluster randomized controlled trial. *Scand J Work Environ Health*.

[19] Paukkunen M, Karppinen J, Öberg B (2025). Cost analysis comparing guideline-oriented biopsychosocial management to usual care for low-back pain: a cluster-randomized trial in occupational health primary care. Scand J Work Environ Health.

[20] Paukkunen MT, Holopainen R, Öberg B (2025). Capabilities, opportunities and motivations in implementing guideline-oriented biopsychosocial low back pain management: perceptions of occupational healthcare professionals after an educational intervention. BMC Health Serv Res.

[21] Overmeer T, Boersma K, Denison E (2011). Does teaching physical therapists to deliver a biopsychosocial treatment program result in better patient outcomes? A randomized controlled trial. Phys Ther.

[22] Fritz J, Overmeer T (2023). Do Physical Therapists Practice a Behavioral Medicine Approach? A Comparison of Perceived and Observed Practice Behaviors. Phys Ther.

[23] Stevenson K, Lewis M, Hay E (2006). Does physiotherapy management of low back pain change as a result of an evidence-based educational programme?. J Eval Clin Pract.

[24] van Trijffel E, A B Oostendorp R, Elvers JWH (2019). Routinely collected data as real-world evidence for physiotherapy practice. Physiother Theory Pract.

[25] Hemkens LG, Contopoulos-Ioannidis DG, Ioannidis JPA (2016). Routinely collected data and comparative effectiveness evidence: promises and limitations. CMAJ.

[26] Sherman RE, Anderson SA, Dal Pan GJ (2016). Real-World Evidence - What Is It and What Can It Tell Us?. N Engl J Med.

[27] Karppinen J, Simula AS, Holopainen R (2021). Evaluation of training in guideline-oriented biopsychosocial management of low back pain in occupational health services: Protocol of a cluster randomized trial. *Health Sci Rep*.

[28] (2026). Occupational healthcare Helsinki: ministry of social affairs and health. https://stm.fi/en/occupational-health-care.

[29] O’Sullivan PB, Caneiro JP, O’Keeffe M (2018). Cognitive Functional Therapy: An Integrated Behavioral Approach for the Targeted Management of Disabling Low Back Pain. Phys Ther.

[30] (2001). International classification of functioning, disability and health: ICF.

[31] Schröder K, Öberg B, Enthoven P (2023). Improved adherence to clinical guidelines for low back pain after implementation of the BetterBack model of care: A stepped cluster randomized controlled trial within a hybrid type 2 trial. Physiother Theory Pract.

[32] George SZ, Zeppieri G (2009). Physical therapy utilization of graded exposure for patients with low back pain. J Orthop Sports Phys Ther.

[33] (2016). Global diffusion of eHealth: making universal health coverage achievable. Report of the third global survey on eHealth.

[34] Nissinen S, Oksanen T, Leino T (2018). Documentation of work ability data in occupational health records. Occup Med (Chic Ill).

[35] (2024). Potilastiedon kirjaamisen yleisopas v 6.0. (General Guide to Documenting Patient Information, version 6.0).

[36] (2012). Potilasasiakirjojen laatiminen ja käsittely. Opas terveydenhuollolle, 4 (Preparation and Processing of Patient Records, A Guide for Healthcare, 4).

[37] Amell T, Kumar S (2001). Work-related musculoskeletal disorders: design as a prevention strategy. A review. J Occup Rehabil.

[38] Uronen L, Heimonen J, Puukka P (2017). Health check documentation of psychosocial factors using the WAI. Occup Med (Chic Ill).

[39] Scholte M, van Dulmen SA, Neeleman-Van der Steen CWM (2016). Data extraction from electronic health records (EHRs) for quality measurement of the physical therapy process: comparison between EHR data and survey data. BMC Med Inform Decis Mak.

[40] Toftdahl AKS, Pape-Haugaard LB, Palsson TS (2018). Collect Once - Use Many Times: The Research Potential of Low Back Pain Patients’ Municipal Electronic Healthcare Records. Stud Health Technol Inform.

[41] (2001). Occupational health care act.

[42] (1992). Act on the status and rights of patients.

[43] (1999). Personal data act (henkilötietolaki).

[44] (2010). Health care act.

[45] (2018). Data protection act.

[46] Khanji C, Schnitzer ME, Bareil C (2019). Concordance of care processes between medical records and patient self-administered questionnaires. BMC Fam Pract.

[47] Weiskopf NG, Bakken S, Hripcsak G (2017). A Data Quality Assessment Guideline for Electronic Health Record Data Reuse. EGEMS (Wash DC).

[48] Kahn MG, Callahan TJ, Barnard J (2016). A Harmonized Data Quality Assessment Terminology and Framework for the Secondary Use of Electronic Health Record Data. EGEMS (Wash DC).

[49] Franklin JM, Schneeweiss S (2017). When and How Can Real World Data Analyses Substitute for Randomized Controlled Trials?. Clin Pharma and Therapeutics.

[50] An D, Lim M, Lee S (2025). Challenges for Data Quality in the Clinical Data Life Cycle: Systematic Review. *J Med Internet Res*.

[51] Steichen O, Daniel-Lebozec C, Charlet J (2006). Use of electronic health records to evaluate practice individualization. AMIA Annu Symp Proc.

[52] Liaw S-T, Guo JGN, Ansari S (2021). Quality assessment of real-world data repositories across the data life cycle: A literature review. J Am Med Inform Assoc.

[53] (2025). The kela statistical yearbook.

[54] Simpson P, Holopainen R, Schütze R (2024). Becoming confidently competent: a qualitative investigation of training in cognitive functional therapy for persistent low back pain. Physiother Theory Pract.

[55] Synnott A, O’Keeffe M, Bunzli S (2015). Physiotherapists may stigmatise or feel unprepared to treat people with low back pain and psychosocial factors that influence recovery: a systematic review. J Physiother.

[56] Mess SA, Mackey AJ, Yarowsky DE (2025). Artificial Intelligence Scribe and Large Language Model Technology in Healthcare Documentation: Advantages, Limitations, and Recommendations. Plast Reconstr Surg Glob Open.

