# Supplementary material for: Where is the patient in the records? Evaluating physiotherapists’ first visit in occupational health primary care pathway for low back pain
Source: BMJ Open Qual. 2026 Feb 26;15(1):e003900. doi: 10.1136/bmjoq-2025-003900 (PMC12959044; doi:10.1136/bmjoq-2025-003900)
Supplement: online supplemental file 1 [file bmjoq-15-1-s001.pdf]

Appendix Table 1. Researcher's evaluation tool to examine physiotherapists' clinical practice in light of their electronic patient record (EPR) documentation.
The tool was developed for study-specific purposes. The instruction was to mark "Yes" if any mention of the item/dimension in question in the EPR was found, "No" if it was not documented at all. Orange shadings further visualize the agreed scorings for the dimensions of assessment and treatment quality criteria.

NAME OF THE UNIT:	PATIENT ID:	Gender: 1 Male / 2 Female / 3 Other
Name of the PT:	Date of patient recruitment:	Age:
Date of PT visit:	Date of control check-ups:	o Visit at clinic o Remote
**ANAMNESIS**		
**BASELINE STATUS**		
Occupation:	Physical workload: o Light o Medium o Heavy	
Work situation: Working: o Full-time o Part-time / Not working: o Short-term sick leave o Long-term sick leave (<2 weeks)		
Current work modifications:		
LBP diagnosis ICD10:	ICP2-code:	1 Non-specific / 2 Specific
Comorbidities:		Number of comorbidities:
**PAIN CHARACTERISTICS**	**Patient´s LBP history taken** [27]	**o Yes o No**
	Time course [27]: 1 Acute / 2 Subacute / 3 Recurrent / 4 Persistent / 5 NA	
	Pain area [27]: 1 Localised / 2 Widespread / 3 NA	
	Progression [27]: 1 Improving / 2 Stable / 3 Deteriorating / 4 NA	
	Number of relapses:	Time of relapses:
	Red flags documented	o Yes o No
**PERSON-CENTRED ASPECTS**	**Person-centred aspects identified** [27]	**o Yes o No**
	Patient´s values documented [27]	o Yes o No
	Patient´s resources documented [27]	o Yes o No
	Patient´s treatment expectations documented [27]	o Yes o No
	Patient´s goals documented [27]	o Yes o No
**FUNCTIONAL CAPACITY**	**Functional capacity documented** [27]	**o Yes o No**
	Pain-related functional behaviours identified* [27]	o Yes o No
	Activity limitation documented [27]	o Yes o No
**WORKABILITY**	**Workability documented** [27]	**o Yes o No**
**ASSESSMENT**		
**STEP I - Multidimensional assessment**		
Cross X all types of assessments documented		Notes of the evaluation
**BIOLOGICAL**	**PHYSICAL ASSESSMENT**	o Yes o No
	o Physical examination* [27]	
	o Range of motions	
	o Provocative spinal movement directions* [27]	
	o Gait	
	o Posture	
	o Motor control	
	o Patient specific functions* [27]	
	o Pain-related physical behaviours* [27]	
	o Autonomic arousal* [27]	
	o Muscle strength	
	o Muscle activation	
	o Physical loading demands [27]	
	o Tissue hypersensitivity [27]	
	o Relationship between physical aspects and pain responses [27]	
	o Other:	
	**HEALTH-RELATED ASPECTS**	o Yes o No
	o Activity and/or sedentary lifestyle [27]	
	o Weight / Obesity [27]	
	o Smoking habits [27]	
	o Nutrition	

	o Sleep deficit [27]	
	o General condition [27]	
	o Pain medication	
	o Psychotropic drugs	
	o Alcohol abuse	
	o Other:	
**PSYCHOLOGICAL**	**COGNITIVE ASPECTS**	o Yes o No
	o Pain-related worries [27]	
	o Coping [27]	
	o Pain catastrophizing [27]	
	o Hypervigilance [27]	
	o Relationship between cognitive stressors and pain responses [27]	
	o Other:	
	**EMOTIONAL ASPECTS**	o Yes o No
	o Distress [27]	
	o Low mood [27]	
	o Fear of physical activity or re-injury [27]	
	o Anxiety feelings [27]	
	o Relationship between emotional stressors and pain responses [27]	
	o Other:	
**SOCIAL**	**SOCIAL ASPECTS**	o Yes o No
	o Life stress events [27]	
	o Social support at home [27]	
	o Socio-economic factors [27]	
	o Social isolation [27]	
	o Cultural factors [27]	
	o Several visits to various practitioners [27]	
	o Complaints or seeking compensation [27]	
	o Relationship between social stressors and pain responses [27]	
	o Other:	
	**WORK-RELATED ASPECTS**	o Yes o No
	o Workplace issues [27]	
	o Work modifications [27]	
	o Self-perceived ability to work [27]	
	o Fear of not coping with work demands [27]	
	o Work community support [27]	
	o Relationship between work stressors and pain responses [27]	
	o Other:	

**TREATMENT**		
**FIRSTLINE TREATMENTS**		
**STEP II -Treatment aligned with low-risk for work disability**		
Use of a risk stratification tool [27]		o Yes o No
o SBT (score __________)	o ÖMPSQ-SF (score _________)	o Other:
Information / education on pain [27]		o Yes o No
Discussion of imaging / use of patient educational booklet “Understanding LBP” [27]		o Yes o No
Advice on return-to-activities (incl. work) [27]		o Yes o No
o Advice to stay active and/or return to normal physical activities		o Advice to avoid physical activities
Pain management strategies [27]		o Yes o No
Addressing healthy lifestyle behaviours [27]		o Yes o No
o Sleep hygiene	o Regular physical activity [27]	o Relaxation / Stress reduction [27]
o Dietary advice	o Substance abandonment	o Other:

**STEP III - Treatment aligned with medium-risk for work disability**		
Use of exercise [27]		o Yes o No
Physical activity based on patient preference and accessibility [27]		o Yes o No
Therapeutic exercise [31]		o Yes o No
o Postural training [31]	o Muscle strengthening training [31]	o Range of movement training [31]
o Postural control training [31]	o Muscle endurance training [31]	o Pelvic floor training [31]
o Core exercise [31]	o Cardiovascular training [31]	o Balance training [31]
o Relaxation training [31]	o Other:	
Reducing pain related fear of movement [27]		o Yes o No
Graded exposure to patient-specific functions / threatening activities* [27]		o Yes o No
Modifying pain-related functional behaviours* [27]		o Yes o No
**STEP IV - Treatment aligned with high-risk for work disability**		
Planned behavioural strategies		o Yes o No
o Psychologically informed care [27]	o Cognitive functional therapy [29]	o Psychophysical physiotherapy
o Mindfulness	o Supportive conversation	o Other:
Relapse plan [27]		o Yes o No
Planned work-related activities		o Yes o No
o Contact with supervisor during visit		o Work modifications [27]
o Keep contact with work during sick leave		o Activity pacing
o Information /education on work-related activities		o Physical activities at work
o Recommendation for assistive devices		o Other:
Goal-oriented treatment plan [27]		o Yes o No
Treatment Plan	Treatment plan [27]	o Yes o No
	o Clear link between anamnesis, assessment and treatment plan	
	Notes of the evaluation	
Treatment Goal	Treatment goal [27]	o Yes o No
	o Short-term	o Long-term
	Goal based on:	
	o Collaboration between therapist and patient [27]	
	o Activities related to patient´s valued goals related [27]	
	o Physiotherapists´ examination	
	o General level LBP rehabilitation	
	o Other:	
	Notes of the evaluation	
**SECOND LINE TREATMENTS**		
**MANUAL THERAPY (used or adviced to)**		
o Joint mobilisation [31]	o Stretching [31]	o Traction [31]
o Joint manipulation [31]	o Nerve mobilisation [31]	o Fascia mobilisation / manipulation [31]
o Massage [31]	o Trigger point pressure [31]	o Other:
**PHYSICAL MODALITIES (used or adviced to)**		
o Heat [31]	o Orthosis [31]	o Taping [31]
o Acupuncture [31]	o TENS [31]	o Other:
Notes of the evaluation		

EPR = electronic patient records; ICD10 = International Classification of Diseases, 10th Revision; ICPC2 = International Classification of Primary Care, 2nd edition; ID = identifier; LBP = low back pain; NA = not assessed; PT = physiotherapist; SBT = STarT Back Tool; TENS = transcutaneous electronic nerve stimulation; ÖMPSQ-SF = Örebro Musculoskeletal Pain Screening Questionnaire

*Documenting patient perceived **pain-related functional behaviours** i.e. communicative or overprotective behaviours such as avoidance of activities
Performing **physical examination** e.g. neurological examination, sit-to-stand, bending, squatting
Assessing **provocative spinal movements** i.e. directional pain responses
Assessing **patient specific functions** i.e. activities the person was unable to perform or found them difficult
Observing **pain-related physical behaviours** i.e. slow guarded movement, avoidance, breath holding, propping with hands, limping, compulsive stretching or use of braces
Observing **autonomic arousal** e.g. rapid superficial breathing, sweating, agitation
Performing **graded exposure** to patient-specific functions / threatening activities i.e. guiding pain and movement control strategies to build confidence for people to re-engage in valued activities [29]

**Modifying pain-related functional behaviours** i.e. identifying and practising alternative ways to do activities and letting go of unhelpful protective habits e.g. moving very cautiously, avoiding activities, holding breath, using compensatory upper limb support to offload during tasks, limping, repetitively stretching to control symptoms and relying on braces or overusing external supports when not needed.

Scoring logic for assessment quality criteria
**Step I - Multidimensional assessment**<br>Criteria: All bio-psycho-social dimensions documented<br>1. Assessing biological, psychological and social dimensions of low back pain
Scoring logic for treatment quality criteria
**Step II - Treatment aligned with low-risk for work disability**<br>Criteria: ≥50% of items 1-5 are documented<br>1. Using risk stratification tool<br>2. Giving patient education<br>3. Advising to stay active<br>4. Discussing pain management strategies<br>5. Addressing lifestyle behaviours
**Step III - Treatment aligned with medium-risk for work disability**<br>Criteria: Step II criteria fulfilled, and ≥50% of items 6-7 are documented<br>6. Using exercise interventions<br>7. Reducing movement-related fear by modifying pain-related behaviours or using graded exposure.
**Step IV - Treatment aligned with high-risk for work disability**<br>Criteria: Step III criteria fulfilled, and at least 2 out of 3 items 8-10 are documented<br>8. Using behavioural interventions<br>9. Using work-related interventions and supporting social engagement<br>10. Making a goal-oriented treatment plan

Appendix Figure 1. Biopsychosocial guideline used in the training

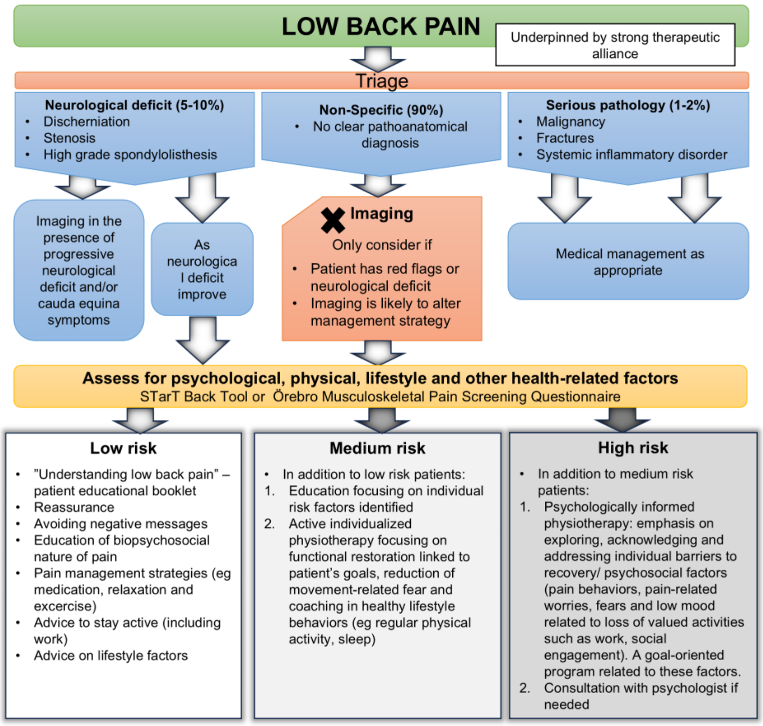

Appendix Figure 2. Multidimensional assessment (IG=intervention group; CG=control group)

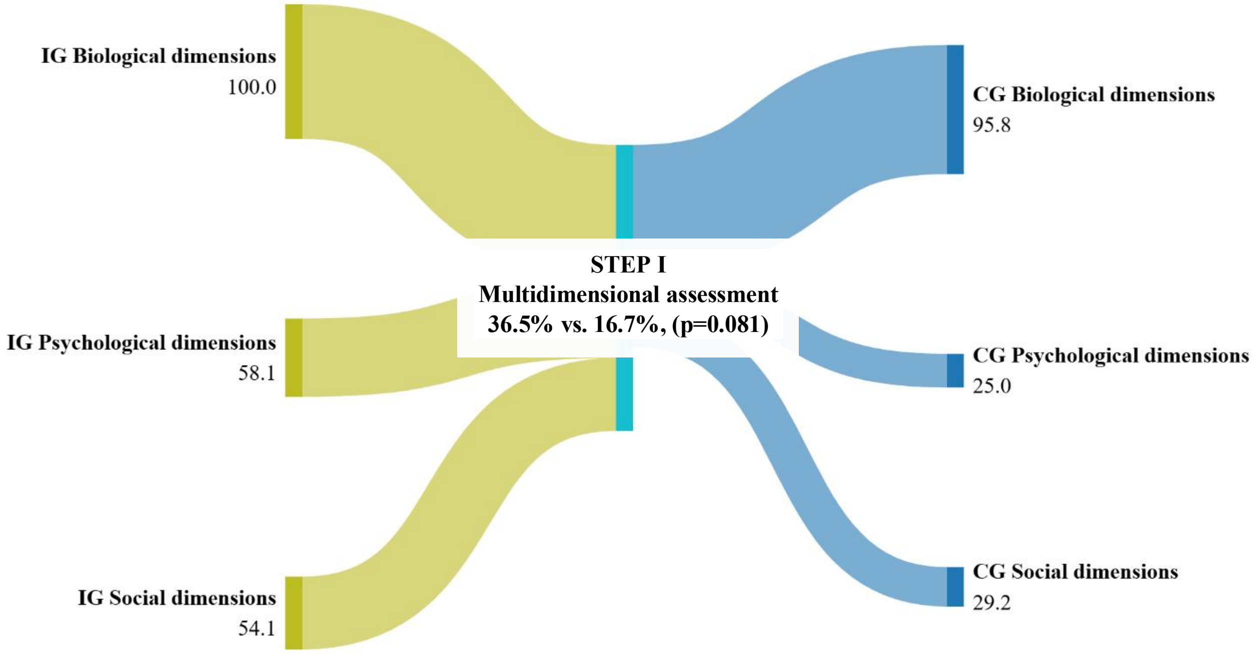

Footnote. Multidimensional assessment: all three dimensions (biological psychological and social) documented in the EPR in the intervention arm (left) and the control arm (right side)
